# Supplementary material for: Global Warming, Advancing Bloom and Evidence for Pollinator Plasticity from Long-Term Bee Emergence Monitoring
Source: Insects. 2021 May 16;12(5):457. doi: 10.3390/insects12050457 (PMC8155920; doi:10.3390/insects12050457)
Supplement: Supplementary file 1 [file insects-12-00457-s001.zip › insects-1206096-supplementary/insects-1206096-s/insects-1206096-Table S1.pdf]

Annual date for first flowering by select woodland perennial wildflowers from Mohonk Reserve, NY USA, 1970-2006. Days since January 1. Data from Cook *et al.* 2008

Genus	Min. days	Median days	Max days	Range in days
Caltha	101	115	129	28
Erythronium	96	110	125	29
Hedyotis	107	120	135	28
Hepatica	86	105	124	38
Polygala	114	128	139	25
Sanguineria	92	107	118	26
Thalictrum	102	115	127	25
Trillium	108	119	133	25

Annual date for first flowering by select montane perennial wildflowers from Rocky Mountain Biological Lab, Crested Butte, Colorado USA, 1975-2000. Days since January 1. Data provided by David Inouye.

Species	Min. days	Median days	Max days	Range in days
Delphinium barbeyi	162	186	218	56
Heterotheca villosa	168	190	219	51
Mertensia fusiformis	116	140	166	50
