# Supplementary figures and images for: Global Warming, Advancing Bloom and Evidence for Pollinator Plasticity from Long-Term Bee Emergence Monitoring
Source: Insects. 2021 May 16;12(5):457. doi: 10.3390/insects12050457 (PMC8155920; doi:10.3390/insects12050457)

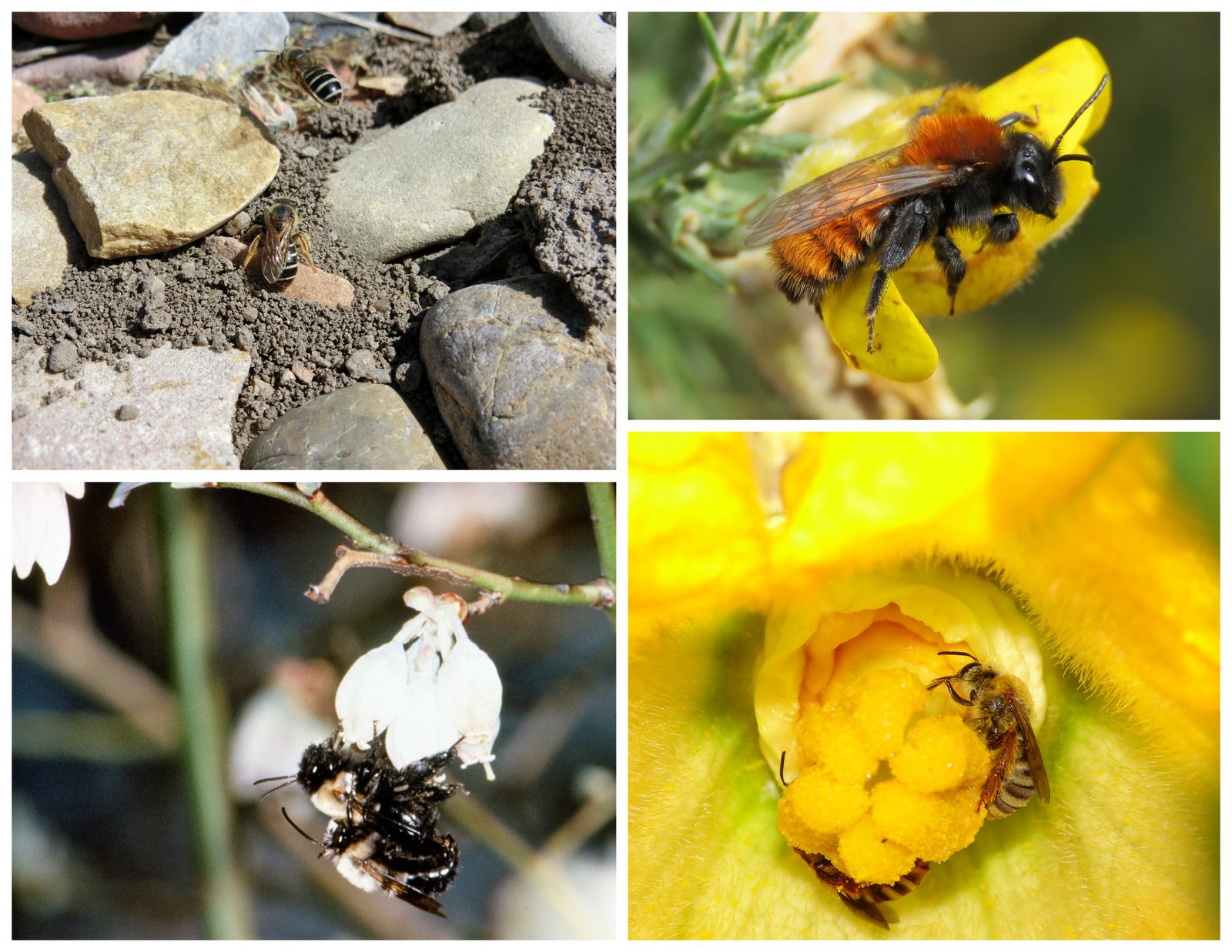

Supplement: Supplementary file 1 [file insects-12-00457-s001.zip › insects-1206096-supplementary/insects-1206096-s/Figure/Suppl.Fig S1 BeesCane.jpg]

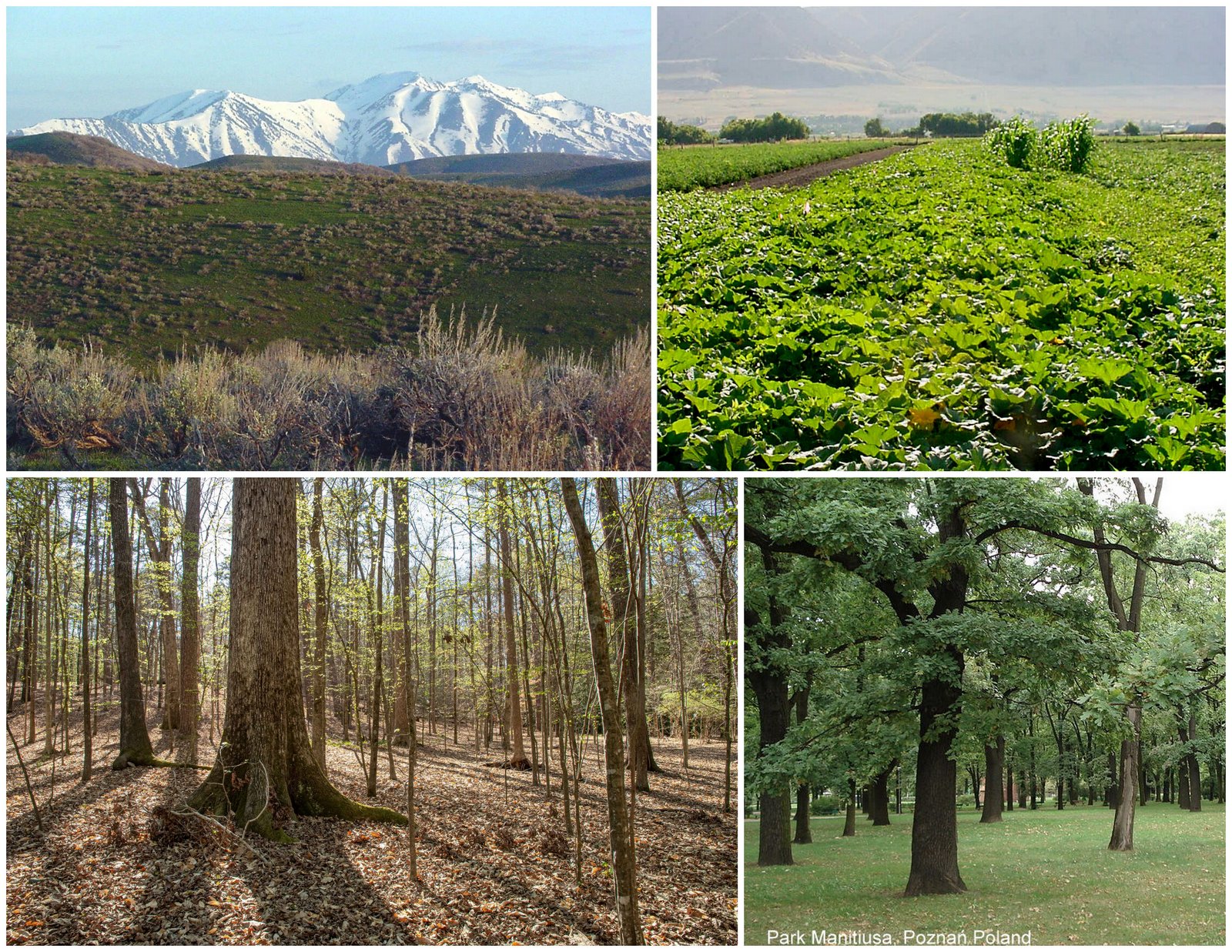

Supplement: Supplementary file 1 [file insects-12-00457-s001.zip › insects-1206096-supplementary/insects-1206096-s/Figure/Suppl.Fig, S2 HabitatsCane.jpg]
